# Post-seismic indoor radon exposure in ground-floor gyms: amplified health risk during physical exercise

**DOI:** 10.3389/fpubh.2026.1814113

**Published:** 2026-05-20

**Authors:** Serdar Baler, Rabia Hurrem Ozdurak Singin, Tuba Denizci, Eda Akkiz Agascioglu

**Affiliations:** 1 Department of Mining and Mining Extraction (Drilling Technology Program), Hekimhan Mehmet Emin Sungur Vocational School, Malatya Turgut Özal University, Malatya, Türkiye; 2 Department of Exercise and Sport Sciences, Faculty of Health Sciences, Malatya Turgut Özal University, Malatya, Türkiye; 3 Independent Researcher, Çorum, Türkiye; 4 Department of Exercise and Sport Sciences, Faculty of Sport Sciences, Istanbul Rumeli University, Istanbul, Türkiye

**Keywords:** disaster recovery, earthquakes, exercise physiology, indoor radon, radiation dose, lung cancer risk, radon

## Abstract

Earthquakes trigger geophysical changes that enhance the release and upward migration of radon-222, potentially elevating indoor radon concentrations in ground-contact buildings well above pre-seismic baseline levels during disaster recovery phases. Although radon carcinogenesis, earthquake-related radon anomalies, and exercise physiology have each been extensively studied in isolation, their convergence in high-occupancy ground-floor gymnasiums has not previously been examined. This paper advances and evaluates the following hypothesis: post-seismic indoor radon accumulation in ground-floor gymnasiums, when combined with exercise-induced increases in pulmonary ventilation, produces internal radiation doses that substantially exceed those predicted by standard concentration-based radon reference levels, representing a disproportionate and under-recognized lung cancer risk for physically active populations during earthquake recovery. To evaluate this hypothesis, we synthesize evidence across earthquake geophysics, exercise physiology, and radon dosimetry. Pre-earthquake indoor radon concentrations of 50–300 Bq/m^3^ reflect documented baseline levels; post-earthquake concentrations of 500–1,000 Bq/m^3^ represent plausible elevations supported by documented post-seismic soil-gas and groundwater anomalies and earthquake-induced building envelope damage. Three user profiles are modeled: a casual visitor (control; dose conversion factor, DCF = 6.9 nSv/(Bq·h·m^−3^); 130–260 h/y), a recreational gym user (DCF = 11 nSv/(Bq·h·m^−3^); 130–260 h/y), and an elite endurance athlete (DCF = 14 nSv/(Bq·h·m^−3^); 800–1,200 h/y). Vigorous exercise increases minute ventilation 5–15-fold and shifts breathing to predominantly oral, amplifying radon progeny inhalation dose approximately 2-fold relative to rest at the same concentration. Model-based illustrations indicate that elite athletes training at post-earthquake concentrations of 500–1,000 Bq/m^3^ may accumulate estimated annual effective doses of 5.6–16.8 mSv/y, substantially exceeding average annual natural background radiation of 1.0–1.8 mSv/y. Session duration and exercise intensity are identified as immediately modifiable risk factors, with 30-min session reductions capable of halving annual dose for recreational users. These findings suggest that standard concentration-based radon guidelines substantially underestimate lung cancer risk for physically active populations in post-earthquake environments because they do not account for exercise-induced physiological amplification of dose. Proactive post-earthquake radon screening in ground-floor gymnasiums, activity-specific temporary exposure guidance, and integration of radon-resistant measures into seismic building codes represent cost-effective and actionable mitigation strategies.

## 1 Introduction

Radon-222, a naturally occurring radioactive gas originating from the uranium decay series within soil and rock, is recognized as the second leading cause of lung cancer worldwide after tobacco smoking (1). Radon enters buildings primarily through pressure-driven soil gas transport, a process governed by soil permeability, foundation integrity, and pressure differentials between the subsurface and indoor environments (1). Radon gas concentration is internationally expressed in becquerels per cubic meter (Bq/m^3^), while some agencies, such as the U.S. Environmental Protection Agency (EPA), also use picocuries per liter (pCi/L), with a practical conversion of 1 pCi/L ≈ 37 Bq/m^3^ (2, 3). Radon exposure remains a significant public health concern, and concentration thresholds such as 200 Bq/m^3^ are widely used in risk assessment and mapping studies (4, 5).

The principal health hazard associated with radon exposure arises not from the inert gas itself but from its short-lived solid decay products, termed radon progeny, including Polonium-218 (Po-218), Lead-214 (Pb-214), Bismuth-214 (Bi-214), and Polonium-214 (Po-214). Upon inhalation, these alpha-emitting radionuclides deposit on the bronchial and bronchiolar epithelium, delivering high linear energy transfer (high-LET) radiation over short path lengths of approximately 40–70 μm. Seismic events profoundly disrupt subsurface radon dynamics (6). Crustal strain accumulation, microfracturing, dilatancy, and earthquake-induced groundwater perturbations enhance radon emanation from radium-bearing minerals and facilitate upward migration through increased permeability and advective transport (7, 8). These processes frequently generate elevated and sometimes prolonged radon fluxes to the surface, resulting in increased indoor radon concentrations in ground-contact buildings (9–11).

While substantial attention has been devoted to post-earthquake structural damage and immediate physical hazards, subtler degradations in indoor air quality (IAQ)—particularly radon accumulation—remain insufficiently addressed in disaster recovery frameworks. This gap is especially consequential for ground-floor gymnasiums and fitness centers, which typically maintain extensive contact with the ground and often exhibit seismically vulnerable features including slab cracks, sump pits, and utility penetrations that facilitate radon ingress (1, 2). Crucially, these environments are characterized by vigorous physical activity: exercise markedly elevates minute ventilation (VE), often 5–15-fold relative to resting conditions, while shifting breathing from nasal to predominantly oronasal and oral patterns (12–14). This physiological state substantially increases inhalation of airborne radon progeny and enhances their deposition in the lower airways, amplifying internal radiation dose and genotoxic burden (15–18).

Against this background, we advance the following central hypothesis: post-seismic indoor radon accumulation in ground-floor gymnasiums, when combined with exercise-induced increases in pulmonary ventilation, produces internal radiation doses that substantially exceed those predicted by standard concentration-based radon reference levels, representing a disproportionate and under-recognized lung cancer risk for physically active populations during earthquake recovery. This hypothesis rests on three interconnected claims. First, seismic activity can produce sustained indoor radon elevations in ground-contact buildings persisting well beyond the immediate post-seismic period. Second, vigorous exercise amplifies the delivered radiation dose per unit radon concentration through physiological mechanisms—chiefly increased VE and altered airway deposition patterns—that current concentration-based guidelines do not adequately capture. Third, the combination of these two factors generates cumulative annual doses with meaningful lung cancer risk implications under established epidemiological models, identifying physically active individuals as a disproportionately exposed subgroup that current radon risk frameworks fail to recognize.

To evaluate this hypothesis, this paper synthesizes evidence across three interconnected domains: (a) geophysical mechanisms driving earthquake-enhanced radon release (7), (b) exercise physiology processes that amplify inhalation dosimetry and internal radiation dose (13, 17), and (c) established epidemiological and dosimetric risk models quantifying radon-related lung cancer risk (19). Quantitative illustrations use a pre-earthquake baseline range of 50–300 Bq/m^3^, reflecting documented typical indoor radon levels from the global average to the WHO reference level upper bound (1, 4), and a post-earthquake range of 500–1,000 Bq/m^3^, representing plausible sustained post-seismic indoor concentrations whose evidentiary basis is evaluated in Section 3.

## 2 Methods

### 2.1 Study design

This paper is structured as a hypothesis and theory article synthesizing published evidence across earthquake geophysics, exercise physiology, and radon dosimetry to advance and evaluate the central hypothesis stated in Section 1. No original experimental data were collected. All quantitative illustrations are derived from published models, established dose conversion frameworks, and documented exposure parameters, and are intended to demonstrate order-of-magnitude relationships rather than precise individual dose predictions.

### 2.2 Quantitative illustration framework and unit conventions

All dose illustrations follow a common computational model. Effective dose per session is calculated as:

*Effective Dose (μSv) = C (Bq/m^3^) × DCF (nSv/(Bq·h·m^−3^)) × t (h)/1,000.*

Annual effective dose is calculated as:

*Annual Dose (mSv/y) = C (Bq/m^3^) × DCF (nSv/(Bq·h·m^−3^)) × T (h/y)/10^9^.*

The following unit conventions are applied consistently throughout this paper and all tables: radon concentration in Bq/m^3^; VE in L/min; dose conversion factor (DCF) in nSv/(Bq·h·m^−3^); effective dose per session in μSv; annual effective dose in mSv/y; working level month in WLM/y. These conventions are not varied in any table or figure.

The effective dose concept applied in this paper follows the radiological protection framework established in ICRP Publication 103 (20), which defines the radiation weighting factor for alpha particles as wR = 20, reflecting their substantially higher biological effectiveness relative to photon radiation. Activity-specific dose conversion factors are derived from this framework as operationalized in ICRP Publication 137 (21) for radon progeny inhalation. An equilibrium factor *F* = 0.4 is applied throughout, consistent with ICRP Publication 137 (21) recommendations for mixed indoor environments. Sensitivity is moderate: varying F between 0.3 and 0.5 alters dose estimates by approximately ±25%, which does not affect the directional conclusions. Dose conversion factors are selected according to activity level following the ICRP Publication 137 scaling framework, as summarized in Table 1. This activity-specific approach differs from the single population-averaged value of 9 nSv/(Bq·h·m^−3^) recommended by UNSCEAR (4) for general population exposure assessments. Under the UNSCEAR approach, vigorous exercise dose estimates would be approximately 35% lower; however, the directional finding remains unchanged under either framework. WLM/y values are calculated using 10 mSv/WLM for rest and light activity and 20 mSv/WLM for vigorous exercise, consistent with ICRP Publication 137 reference coefficients (21).

**Table 1:** Dose conversion factors for radon progeny inhalation by activity level (ICRP publication 137, *F* = 0.4).

Activity level	Typical VE (L/min)	DCF (nSv/(Bq·h·m^−3^))	Rationale/references
At rest	6–12	6.9	Standard indoor sedentary conditions; ICRP Publication 137 (21)
Low/light activity	15–25	9–10	Light walking, stretching; slight increase in VE; ICRP Publication 137 (21)
Moderate activity	40–60	10–12	Partial elevation due to increased VE; mixed nasal–oral breathing; ICRP Publication 137 (21)
Moderate–vigorous (Profile 2, recreational)	40–80	11	Intermediate value applied to Profile 2; reflects mixed moderate–vigorous intensity typical of recreational gym use
Vigorous/high-intensity (Profile 3, elite)	80–120+	14	Elevated VE and predominantly oral breathing; substantially altered bronchial deposition; ICRP Publication 137 (21); Marsh et al. (17)

DCF, dose conversion factor in nSv/(Bq·h·m^−3^). VE: minute ventilation in L/min. Values follow ICRP Publication 137 (21) activity-level scaling. The intermediate DCF of 11 nSv/(Bq·h·m^−3^) applied to Profile 2 (recreational user) is interpolated between the moderate (10–12) and vigorous (14) ranges and reflects mixed moderate–vigorous exercise intensity typical of recreational gym use. UNSCEAR (4) recommends a single population-averaged value of 9 nSv/(Bq·h·m^−3^) for general exposure assessments.

### 2.3 Exposure scenarios and radon concentration ranges

Two radon concentration ranges are used throughout this paper. The pre-earthquake baseline range of 50–300 Bq/m^3^ reflects documented typical indoor radon levels: 50 Bq/m^3^ approximates the global average indoor radon concentration (1, 4), while 300 Bq/m^3^ corresponds to the upper limit of the WHO-recommended reference level range (1). This range requires no extrapolation and is directly anchored to published measurement data. The post-earthquake range of 500–1,000 Bq/m^3^ represents plausible sustained post-seismic indoor concentrations; the evidentiary basis for this range is evaluated explicitly in Section 3.2. It must be acknowledged that direct indoor radon measurements in post-earthquake gymnasiums are currently absent from the published literature, and the 500–1,000 Bq/m^3^ range constitutes a hypothesis-building estimate rather than an empirically verified indoor concentration.

Three user profiles are modeled to reflect the realistic range of gymnasium occupants. Profiles 1 and 2 share identical annual exposure hours (130–260 h/y), based directly on WHO physical activity recommendations of 150–300 min/week. This design choice is deliberate: holding time constant between Profiles 1 and 2 while varying exercise intensity and DCF means that any dose difference between these profiles is attributable purely to exercise-induced physiological amplification, providing the clearest possible test of the exercise component of the central hypothesis. Profile 3 demonstrates the compound effect of both higher intensity and substantially higher annual volume. The profiles are defined in Table 2.

**Table 2:** User profiles for quantitative dose illustrations.

Profile	Description	Exercise intensity	VE (L/min)	DCF (nSv/ Bq·h·m^−3^)	Annual exposure (h/y)	Role in analysis
1. Casual visitor (control)	Non-exercising gym occupant: visitor, parent watching training, coach on sideline, or part-time reception staff	Rest/very light	6–12	6.9	130–260	Control group—dose driven by occupancy time alone, no exercise amplification
2. Recreational gym user (general public)	Individuals exercising for health or leisure, consistent with WHO physical activity recommendations of 150–300 min/week	Moderate to vigorous (3–5 sessions/week)	40–80	11	130–260	General public comparator—identical hours to Profile 1, isolating the exercise amplification effect
3. Elite endurance athlete (professional)	Competitive athlete for whom training is the primary professional occupation (e.g., marathon runner, triathlete, professional team sport athlete)	High-intensity vigorous sustained	80–120 + (may exceed 150 at peak)	14	800–1,200	Highest-risk subgroup—compound effect of maximum physiological amplification and maximum annual volume

VE: minute ventilation in L/min. DCF: dose conversion factor in nSv/(Bq·h·m^−3^). Annual exposure hours for Profiles 1 and 2 are matched at 130–260 h/y (derived from WHO physical activity recommendation of 150–300 min/week: lower bound = 150 min/week × 52 weeks/60; upper bound = 300 min/week × 52 weeks/60). Annual exposure for Profile 3 (800–1,200 h/y) reflects documented elite endurance training volumes of approximately 15–23 h/week. DCF values follow ICRP Publication 137 activity-level scaling (21); see Table 4.

### 2.4 Physiological basis for exercise-induced dose amplification

#### 2.4.1 Lung volumes, ventilatory mechanics, and the ventilatory response to exercise

A clear understanding of pulmonary mechanics is essential for interpreting the exercise-induced amplification of radon progeny dose modelled in this paper. Under resting conditions, a healthy adult maintains a tidal volume (VT) of approximately 0.4–0.5 L at a breathing frequency (fB) of 12–15 breaths per minute, yielding a resting VE of approximately 6–9 L/min (12, 22). Total lung capacity (TLC) in healthy adults is approximately 6 L, of which functional residual capacity (FRC) defined as the volume remaining in the lungs at the end of a passive expiration, accounts for approximately 2.5–3.0 L (23). These baseline values define the ventilatory reserve that is progressively recruited during physical activity.

During light physical activity, VE rises to approximately 20–30 L/min, driven primarily by increases in VT rather than breathing frequency. As exercise intensity increases, VT rises until it approaches approximately 50–60% of vital capacity, after which further increases in VE are achieved predominantly through progressive increases in fB (24). At moderate exercise intensities (approximately 50–60% of maximal oxygen uptake, VO₂max), VE commonly reaches 40–60 L/min. At near-maximal effort, VE routinely exceeds 100–120 L/min in healthy sedentary adults, with values exceeding 150 L/min documented in highly trained endurance athletes (12, 13, 24). The progressive increase in VE directly and proportionally increases the mass of airborne radon progeny inhaled per unit time: at 120 L/min relative to 9 L/min at rest, approximately 13 times more air, and proportionally more suspended radon progeny passes through the airways each minute.

The ventilatory response to exercise is regulated through multiple interacting mechanisms. Neurogenic input from locomotor muscles provides an anticipatory drive at exercise onset, while peripheral chemoreceptors and central pattern generators refine the response as metabolic demand increases (13). During heavy exercise in endurance athletes, ventilatory limitation may occur: expiratory flow limitation and dynamic hyperinflation develop when the required VE approaches the mechanical limits of the respiratory system, and operating lung volumes shift upward, reducing inspiratory reserve (23, 24). These constraints become dosimetrically relevant because they alter the distribution of inhaled air within the lung and the regional deposition of radon progeny, as discussed below.

#### 2.4.2 Pulmonary circulation and its interaction with radon gas kinetics

The pulmonary circulation undergoes profound changes during exercise that merit explicit consideration in the context of radon inhalation dosimetry. At rest, cardiac output (Q̇) is approximately 5 L/min, with a mean pulmonary arterial pressure of approximately 14 mmHg (25). During maximal aerobic exercise, cardiac output rises to 20–25 L/min in healthy adults and may exceed 35–40 L/min in elite endurance athletes, achieved through parallel increases in heart rate and stroke volume (25, 26). Pulmonary vascular resistance decreases substantially through the recruitment and distension of pulmonary capillaries, maintaining relatively low pulmonary arterial pressures despite the marked elevation in flow (26).

The ventilation-to-perfusion (VA/Q) ratio, approximately 0.8 at rest, becomes more heterogeneous during heavy exercise, with preferential perfusion of dependent lung zones (22). This heterogeneity modifies the regional distribution of gas exchange and, by extension, the regional uptake of inhaled radon gas into the pulmonary capillary blood. However, it is important to clarify the dosimetric significance of these circulatory changes: radon gas itself contributes negligibly to the radiation dose received by bronchial epithelial cells compared to its solid progeny, which deposit on airway surfaces and deliver alpha radiation locally prior to any systemic distribution. Increased pulmonary blood flow during exercise may marginally accelerate radon gas uptake, thereby slightly reducing the residence time of free radon in the airways and attenuating the very small direct contribution of radon gas to epithelial dose, an effect that slightly counteracts rather than amplifies progeny-mediated dose. The primary dosimetric pathway, namely solid radon progeny deposition on bronchial epithelium, is governed by ventilatory mechanics and breathing route, rather than by pulmonary blood flow per se.

#### 2.4.3 Breathing route transition and its amplification of radon progeny dose

Beyond the volumetric increase in VE, exercise induces a qualitative shift in breathing pattern that further amplifies radon progeny dose independently of concentration. Under resting conditions, nasal breathing predominates in healthy adults, providing effective filtration of inhaled particles through the combined mechanisms of inertial impaction in nasal turbinates, sedimentation in nasal passages, and mucociliary clearance (27). These mechanisms are highly effective for particles with aerodynamic diameters above approximately 1 μm, removing a substantial fraction of attached radon progeny before they can reach the tracheobronchial tree.

As exercise intensity increases and V̇E rises above approximately 35–40 L/min, a threshold corresponding roughly to light-to-moderate exercise intensity, breathing transitions progressively from nasal to oronasal and ultimately to predominantly oral (14). At VE values exceeding 60 L/min, oral breathing predominates almost exclusively (14, 27). This transition bypasses nasal filtration entirely, producing two important dosimetric consequences: a substantially greater fraction of inhaled radon progeny reaches the tracheobronchial tree, and higher linear airflow velocities in the larger airways increase inertial impaction of progeny on the bronchial epithelium, which is the primary target tissue for radon-induced carcinogenesis (18, 28).

Radon progeny exist in the atmosphere in two physical states relevant to deposition: the unattached fraction (electrostatically charged, effective diameters of 0.5–5 nm) and the attached fraction (associated with ambient aerosol particles, effective diameters of 0.1–1 μm). Because nasal bypass during vigorous exercise increases penetration of both fractions to the tracheobronchial tree, and because higher airflow velocities favour inertial impaction of the attached fraction in the bronchi and bronchioles, the combined effect of increased VE and oral breathing dominance substantially elevates bronchial epithelial dose per unit of radon concentration relative to resting conditions (16, 17, 21).

These well-established physiological mechanisms are captured quantitatively in the activity-specific DCFs applied throughout this paper, following the ICRP Publication 137 framework (17, 21): the DCF of 14 nSv/(Bq·h·m^−3^) for vigorous exercise compared to 6.9 nSv/(Bq·h·m^−3^) for rest represents an approximately 2-fold amplification attributable entirely to the physiological differences in respiratory mechanics described in this section. The DCF framework thus provides a single integrated parameter that encapsulates the combined effects of increased VE, oral breathing dominance, and altered bronchial deposition patterns, enabling the dose calculations presented in Table 2, to translate radon concentration directly into activity-specific effective dose at the bronchial epithelium.

### 2.5 Radon progeny deposition, alpha particle path length, and dosimetric boundaries

The health risk associated with indoor radon exposure arises almost exclusively from the short-lived solid radon progeny, principally Po-218 and Po-214, rather than from radon gas itself. Upon inhalation, these electrically charged progeny atoms deposit predominantly in the tracheobronchial region through inertial impaction, gravitational sedimentation, and diffusion. The bronchial and bronchiolar epithelium receives the highest local radiation dose, and the basal and secretory cells of this epithelium are considered the principal targets for radon-induced carcinogenesis (6, 29).

Alpha particles emitted by Po-218 and Po-214 have energies of approximately 6.0 and 7.7 MeV, respectively, and travel a maximum path length of approximately 40–70 μm in tissue—roughly one to two cell layers. This extremely short range means that alpha radiation energy is deposited with very high spatial precision in the immediate vicinity of the decay site, producing dense ionization tracks and complex clustered DNA damage (30–32). Cells beyond approximately 70 μm from a decay site receive essentially no direct alpha irradiation.

Radiation-generated reactive oxygen species (ROS) produced at the bronchial epithelium have extremely short biological half-lives (nanoseconds to microseconds) and travel distances of only nanometers to micrometers in aqueous biological media before reacting with surrounding molecules. Direct ROS-mediated effects on tissues beyond the bronchial epithelium cannot be supported on mechanistic grounds at environmental radon concentrations. Indirect systemic effects are plausible through radiation-induced bystander effects and cytokine-mediated signaling (33), but their magnitude at the dose rates modeled here remains poorly quantified. All dose calculations in this paper are confined strictly to the bronchial epithelium, consistent with established ICRP dosimetric models.

## 3 Mechanisms of post-seismic radon release and indoor accumulation

### 3.1 Radon emanation and migration enhanced by seismic stress

The release of radon gas following seismic events results from a range of physical and mechanical alterations within the subsurface. Radon-222 is continuously produced by radioactive decay of radium-226 within mineral grains, and its transport to the surface is governed by two key processes: emanation and migration (1).

Emanation involves the release of radon atoms from radium-bearing minerals into surrounding pore spaces. Seismic activity induces crustal strain, micro-fracturing, and structural disruption at grain boundaries, increasing the effective surface area of these minerals and creating fresh escape pathways. The source strength of radon in soils and bedrock is thereby enhanced, particularly in faulted and fractured geological settings (8, 34–37).

Migration refers to the subsequent transport of radon through interconnected pore networks and fractures toward the atmosphere, primarily via advection driven by pressure gradients. Dilatancy processes induce fracturing and volumetric expansion, increasing bulk permeability (8, 37–40). Dynamic shaking perturbs soil columns and groundwater systems, liberating trapped gas pockets and effectively pumping radon-bearing soil gas upward (35, 37, 41). Coseismic permeability enhancement along fault zones can persist well beyond the mainshock, supporting sustained increases in radon flux (36, 42, 43). The synergy between enhanced emanation and more efficient migration produces a pronounced and often prolonged surge in surface radon flux, with post-seismic elevations documented to persist for months to years in some geological settings (11, 42).

### 3.2 Post-seismic radon infiltration into buildings: the logical bridge from soil-gas anomalies to indoor concentrations

Elevated radon flux from the subsurface increases the driving force for radon infiltration into buildings through pressure differentials between soil gas and indoor air. This process is strongly influenced by soil permeability, foundation integrity, and building–soil pressure gradients (44, 45).

Under non-seismic conditions, typical indoor radon concentrations in ground-floor buildings range from approximately 50 Bq/m^3^ at the global average to 300 Bq/m^3^ at the upper bound of WHO reference levels, reflecting normal pressure-driven soil-gas ingress through intact foundations (1, 4). Following seismic events, two compounding mechanisms elevate this baseline. First, the subsurface radon flux increases substantially: post-seismic soil-gas and groundwater radon anomalies of 2- to 10-fold above pre-seismic levels have been documented in affected geological settings (11, 34, 35). Second, earthquake-induced structural damage to building foundations, including slab cracking, displacement of utility penetrations, and disruption of sub-slab membranes, substantially increases effective entry area and reduces resistance to soil-gas ingress (1, 2).

Direct evidence for post-seismic indoor radon elevation is available from the 2016–2017 Gyeongju–Pohang earthquake sequence, in which measurable indoor radon increases were documented in buildings proximate to active fault zones (46). Long-term investigations following the 2016 Kumamoto earthquake documented groundwater and soil-gas radon levels remaining elevated for more than 2 years after the mainshock (11), and the 2011 northern Wakayama earthquake was associated with anomalous atmospheric radon increases persisting for several months (47, 48). The 2025 Mw 7.7 Myanmar earthquake documented a pronounced pre-seismic radon anomaly persisting for approximately 109 days (8), further reinforcing the plausibility of sustained post-seismic elevations in regions experiencing prolonged strain redistribution.

Taken together, a post-seismic indoor radon range of 500–1,000 Bq/m^3^ in ground-floor buildings with earthquake-damaged foundations represents a plausible 2–3-fold elevation above the upper pre-seismic reference bound, consistent with the combined effect of enhanced subsurface flux and compromised building envelope integrity. Gymnasiums and fitness centers are particularly vulnerable, typically occupying ground-floor spaces with extensive slab contact, multiple utility penetrations, sump pits, and drainage channels that represent preferential entry pathways even under normal conditions (1, 2). It must be acknowledged, however, that direct indoor radon measurements in post-earthquake gymnasiums are currently absent from the published literature, and the 500–1,000 Bq/m^3^ range constitutes a hypothesis-building estimate rather than an empirically verified indoor concentration.

## 4 Genotoxic mechanisms of radon and progeny

The primary health hazard associated with radon inhalation does not originate from the inert gas itself, but from its short-lived solid decay products, particularly Po-218 and Po-214 (29, 49). Upon inhalation, these alpha-emitting radionuclides deposit on the bronchial and bronchiolar epithelium, delivering high-LET radiation over path lengths of approximately 40–70 μm. The high-LET of alpha particles produces dense ionization tracks causing complex molecular damage (30–32). For radiological protection, the ICRP expresses radon progeny exposure as effective dose (mSv/y) using activity-adjusted DCF values in nSv/(Bq·h·m^−3^), accounting for internal alpha irradiation of the bronchial epithelium (17, 21, 50).

When an alpha particle track intersects a cell nucleus, it induces severe and spatially clustered DNA damage, most notably double-strand breaks (DSBs) and complex lesions referred to as Multiply Locally Damaged Sites (MLDS) (21, 30, 31, 51–54). Non-homologous end joining (NHEJ) serves as the primary DSB repair mechanism but is intrinsically error-prone, potentially generating deletions, insertions, or chromosomal rearrangements (51–53, 55). Homologous recombination offers higher fidelity but can be overwhelmed by the density of alpha-induced lesions (30, 56). The tumour suppressor protein p53 orchestrates cell-cycle arrest and apoptosis; p53 dysfunction permits survival of genomically unstable cells, promoting malignant transformation (57–59).

Robust epidemiological evidence from occupational cohorts of underground miners confirms these mechanisms, with significantly elevated frequencies of chromosomal aberrations (60, 61) and mutations at critical loci including HPRT, TP53, and KRAS (62–66). Comparable genotoxic endpoints—including DNA strand breaks and chromosomal damage—have been observed in residential populations exposed to indoor radon concentrations ≥300 Bq/m^3^ (67, 68). Key biomarker findings are summarized in Table 3. In addition to direct DNA damage, alpha radiation induces radiolysis of water molecules, generating ROS that contribute to oxidative base damage, point mutations, and secondary genomic instability (31, 33, 51, 69). As established in Section 2.5, these effects are spatially confined to the bronchial epithelium and do not support claims of direct systemic effects at environmental exposure concentrations.

**Table 3:** Summary of genotoxic biomarkers associated with radon exposure.

Biomarker/effect	Study model	Key findings	Exposure level	References
Chromosomal aberrations	Human (peripheral blood lymphocytes)	Significant increases in dicentric and acentric fragments; elevated chromosomal abnormalities at higher radon doses	0–120 mGy; elevated indoor radon	(61)
	Human (miners)	Increased frequency of chromosomal aberrations in uranium and underground miners	High radon levels	(60, 61)
	Human (residents of high-radon areas)	No significant increase in unstable chromosomal aberrations reported in some populations	High indoor radon	(79)
	Rat (*in vivo*)	Increased chromosomal and chromatid breaks in hematopoietic stem/progenitor cells	13.01–65.05 WLM	(90)
Micronucleus (MN)	Human (peripheral blood lymphocytes, buccal cells)	Dose-dependent increase in micronucleus frequency; elevated binucleated lymphocytes in high-radon residents	Increased radon dose; high indoor radon	(61, 80)
	Human (occupational exposure)	Significant positive correlation between micronucleus frequency and occupational exposure duration	Low-dose radiation	(81)
Sister chromatid exchange (SCE)	Human—*in vitro* (normal human lung fibroblasts)	Significant increase in SCE frequency in human lung fibroblasts following alpha particle exposure; both direct and indirect SCE induction pathways relevant to radon progeny exposure	Low-dose alpha particle irradiation	(91)
Gene mutations (HPRT, GPA)	Human (lung tumour tissue, bronchial epithelial cells)	TP53 mutations and K-ras mutations in radon-associated lung cancers from uranium miners; p53 codon 213 polymorphism; HPRT deletions; KRAS overexpression with chronic radon exposure	Chronic; uranium miners	(61–66)
	Human (in vitro, human lymphoblastoid TK6 cells)	Induced mutant fractions at HPRT and TK loci; high frequency of partial or complete deletions in the HPRT gene	Experimental radon exposure	(92)

WLM, working level month; GPA, glycophorin A; HPRT, hypoxanthine-guanine phosphoribosyltransferase.

## 5 Synergistic amplification of radon progeny dose by exercise intensity and duration

The genotoxic burden from radon progeny is critically dependent on inhaled air volume and deposition patterns. Vigorous exercise amplifies both: VE increases 5–15-fold and breathing shifts to predominantly oral, enhancing progeny delivery to deep airways (12–14). Table 4 presents the complete dose matrix across all three user profiles and all four radon concentration scenarios, including a per-session chest X-ray equivalent column provided for public risk communication. The exercise-to-rest amplification at identical radon concentration is approximately 2.0-fold, reflecting the ratio of activity-specific DCF values (14/6.9 ≈ 2.0). This physiological amplification compounds with concentration elevation: comparing a casual visitor at pre-earthquake baseline (50 Bq/m^3^, rest: 0.35 μSv per session hour, equivalent to approximately 0.02 PA chest radiographs) with an elite athlete exercising vigorously in a severe post-earthquake environment (1,000 Bq/m^3^, vigorous: 14.0 μSv per session hour, equivalent to approximately 0.70 PA chest radiographs) yields a combined per-hour amplification of approximately 40-fold. It is this compound effect, elevated concentration acting on a physiologically amplified inhalation rate over a much greater annual exposure time, that constitutes the central quantitative argument of this paper.

**Table 4:** Estimated effective dose per 1-h session and annual effective dose—all user profiles × all radon concentration scenarios.

Scenario/radon conc. (Bq/m^3^)	Profile	DCF (nSv/Bq·h·m^−3^)	Effective dose per 1 h session (μSv)	Annual exposure (h/y) lower–upper	Annual effective dose (mSv/y) lower–upper	WLM/y lower–upper
Pre-EQ low 50 Bq/m^3^	1. Casual visitor (control)	6.9	0.34	130–260	0.04–0.09	0.004–0.009
	2. Recreational gym user	11	0.55	130–260	0.07–0.14	0.007–0.014
	3. Elite endurance athlete	14	0.7	800–1,200	0.56–0.84	0.028–0.042
Pre-EQ high 300 Bq/m^3^	1. Casual visitor (control)	6.9	2.07	130–260	0.27–0.54	0.027–0.054
	2. Recreational gym user	11	3.3	130–260	0.43–0.86	0.043–0.086
	3. Elite endurance athlete	14	4.2	800–1,200	**3.36–5.04**	0.168–0.252
Post-EQ moderate 500 Bq/m^3^	1. Casual visitor (control)	6.9	3.45	130–260	0.45–0.9	0.045–0.090
	2. Recreational gym user	11	5.5	130–260	0.72–1.43	0.072–0.143
	3. Elite endurance athlete	14	7	800–1,200	**5.6–8.4**	0.280–0.420
Post-EQ severe 1,000 Bq/m^3^	1. Casual visitor (control)	6.9	6.9	130–260	0.9–1.79	0.090–0.179
	2. Recreational gym user	11	11	130–260	**1.43–2.86**	0.143–0.286
	3. Elite endurance athlete	14	14	800–1,200	**11.2–16.8**	0.560–0.840

Effective dose (μSv/session) = C (Bq/m^3^) × DCF (nSv/(Bq·h·m^−3^)) × 1 h/1,000. Annual effective dose (mSv/y) = C (Bq/m^3^) × DCF (nSv/(Bq·h·m^−3^)) × T (h/y)/10^9^. Equilibrium factor *F* = 0.4 throughout. Pre-earthquake range (50–300 Bq/m^3^): documented typical indoor radon levels (1, 4). Post-earthquake range (500–1,000 Bq/m^3^): plausible post-seismic indoor concentrations as argued in Section 3.2. WLM/y calculated using 10 mSv/WLM for Profiles 1 and 2 and 20 mSv/WLM for Profile 3 (21). Bold annual dose values indicate scenarios where the lower-bound estimate ≥1 mSv/y. All values are illustrative estimates.

For public risk communication purposes only: a single 1-h vigorous exercise session at 500 Bq/m^3^ delivers an estimated effective dose of approximately 7.0 μSv to an elite athlete (Profile 3)—comparable in order of magnitude to approximately one-third of the dose received from a standard postero-anterior chest radiograph [reference dose: 20 μSv (93)]. At 1,000 Bq/m^3^ this value approximately doubles to 14.0 μSv, equivalent to approximately two-thirds of a single chest radiograph. These comparisons are provided solely to convey the order of magnitude of per-session exposure in terms familiar to a non-specialist audience and must not be interpreted as radiobiological equivalence: internal alpha irradiation from radon progeny and external diagnostic X-ray exposure differ fundamentally in radiation type, spatial distribution of energy deposition, tissue geometry, and biological effectiveness.

The dose matrix in Table 5 reveals three important gradients. First, comparing Profiles 1 and 2 at any given concentration demonstrates the exercise amplification effect in isolation: at 500 Bq/m^3^, a recreational user accumulates 0.72–1.43 mSv/y compared to 0.45–0.90 mSv/y for a casual visitor spending identical hours in the same environment, a difference attributable entirely to physiological differences in respiratory mechanics. Second, Profile 3 demonstrates the compound effect of higher intensity and greater annual volume: at 500 Bq/m^3^, the elite athlete range of 5.60–8.40 mSv/y represents 6–9 times the dose received by Profiles 1 and 2. Third, the concentration gradient from pre-earthquake to post-earthquake approximately doubles dose across all profiles, confirming that both the exposure condition and the physiological state of the occupant are independently significant risk factors.

**Table 5:** Session duration and exercise intensity as modifiable risk factors—annual effective dose across all three user profiles at post-earthquake concentrations (500 and 1,000 Bq/m^3^).

Profile	Session duration (min)	Sessions per week	Annual exposure (h/y)	Annual effective dose at 500 Bq/m^3^ (mSv/y)	Annual effective dose at 1,000 Bq/m^3^ (mSv/y)	Implication for risk management
1. Casual visitor (control)	30	3–5	78–130	0.27–0.45	0.54–0.9	Short visits: below 1 mSv/y across all post-EQ scenarios; lowest-risk occupancy pattern
	60	3–5	156–260	0.54–0.9	**1.08–1.79**	Standard visit: remains below 1 mSv/y at moderate post-EQ; approaches reference at severe
	90	3–5	234–390	0.81–1.35	**1.61–2.69**	Extended visit: crosses 1 mSv/y at 1,000 Bq/m^3^; relevant for temporary shelter use
	120	3–5	312–520	**1.08–1.79**	**2.15–3.59**	Prolonged occupancy: exceeds 1 mSv/y at severe post-EQ; shelter-use scenario requires monitoring
2. Recreational gym user	30	3–5	78–130	0.43–0.72	0.86–1.43	Short recreational session: below 1 mSv/y at moderate post-EQ; manageable with ventilation
	60	3–5	156–260	0.86–1.43	**1.72–2.86**	WHO-recommended activity: approaches or crosses 1 mSv/y at post-EQ moderate–severe; ventilation essential
	90	3–5	234–390	**1.29–2.15**	**2.57–4.29**	Extended recreational session: clearly exceeds 1 mSv/y; duration reduction recommended above 500 Bq/m^3^
	120	3–5	312–520	**1.72–2.86**	**3.43–5.72**	Long recreational session: 2–3 × reference level at post-EQ moderate; strong case for session shortening
3. Elite endurance athlete	30	5–6	130–156	0.91–1.09	**1.82–2.18**	Even minimal elite training crosses 1 mSv/y at post-EQ moderate; relocation to outdoor training warranted
	60	5–6	260–312	**1.82–2.18**	**3.64–4.37**	Standard elite session: 2–5 × reference level; temporary outdoor training strongly recommended
	90	5–6	390–468	**2.73–3.28**	**5.46–6.55**	Extended elite session: 3–7 × reference level; unacceptable exposure without structural mitigation
	120	5–6	520–624	**3.64–4.37**	**7.28–8.74**	Long elite session: up to 14 × reference level at severe post-EQ; highest-risk scenario in this model

Annual effective dose (mSv/y) = C (Bq/m^3^) × DCF (nSv/(Bq·h·m^−3^)) × T (h/y)/10^9^. DCF values: 6.9 nSv/(Bq·h·m^−3^) for Profile 1 (rest); 11 nSv/(Bq·h·m^−3^) for Profile 2 (moderate–vigorous); 14 nSv/(Bq·h·m^−3^) for Profile 3 (vigorous). Equilibrium factor *F* = 0.4. Annual exposure hours calculated as: session duration (h) × sessions per week × 52 weeks. Bold values indicate annual effective dose ≥1 mSv/y. Post-earthquake concentrations only are shown as these represent the novel exposure scenario of this paper; pre-earthquake doses are available in Table 5. All values are illustrative estimates.

Table 3 extends this analysis by examining how session duration interacts with exercise intensity across all three profiles under post-earthquake conditions, at four standardized session lengths of 30, 60, 90, and 120 min. This table directly addresses the policy-relevant question of which risk factors are most amenable to immediate modification: session duration and exercise intensity can be reduced through temporary activity-specific guidance without requiring structural intervention.

**Figure 1:**
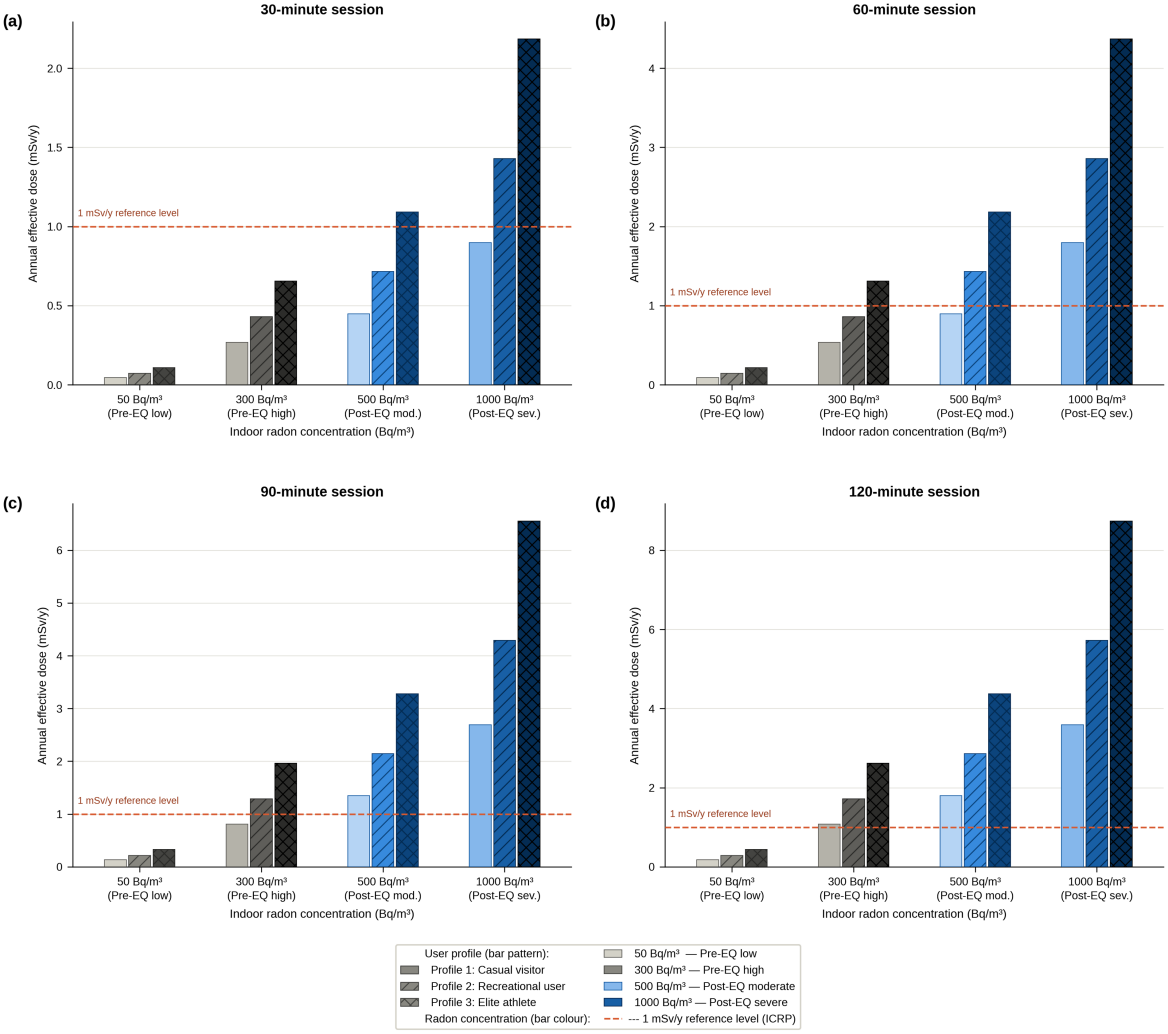
Estimated annual effective dose (mSv/y, upper-bound estimates) by user profile, radon concentration scenario, and session duration. **(a–d)** Correspond to session durations of 30, 60, 90, and 120 min, respectively. X-axis: four radon concentration scenarios spanning the pre-earthquake baseline (50 Bq/m^3^ and 300 Bq/m^3^, grey tones) and post-earthquake range (500 Bq/m^3^ and 1,000 Bq/m^3^, blue tones). Grouped bars: Profile 1 (casual visitor, DCF 6.9 nSv/(Bq·h·m^−3^), mid-grey); Profile 2 (recreational user, DCF 11 nSv/(Bq·h·m^−3^), light blue); Profile 3 (elite athlete, DCF 14 nSv/(Bq·h·m^−3^), dark blue). Annual exposure times (upper bounds): Profiles 1 and 2: 260 h/y; Profile 3: 1,200 h/y. Equilibrium factor *F* = 0.4. Dashed coral line: 1 mSv/y reference level (ICRP, provided for context; radon is an existing exposure situation). All values are illustrative upper-bound estimates; see Section 2.3 for uncertainty discussion.

Figure 1 presents the annual effective dose estimates as a four-panel grouped bar chart, one panel per session duration (30, 60, 90, and 120 min), with x-axis showing the four concentration scenarios and grouped bars for the three profiles. Upper-bound estimates are shown to reflect the maximum plausible exposure for each profile. The reference line at 1 mSv/y illustrates how the combination of duration, intensity, and concentration determines which scenarios exceed this threshold: at 30 min, only Profile 3 at post-earthquake concentrations crosses the line; by 120 min, even recreational users are at or above it across post-earthquake scenarios.

The ICRP reference level of 1 mSv/y for planned exposure situations is included in Figure 1 solely for contextual orientation. It is acknowledged that radon is classified as an existing exposure situation rather than a planned exposure situation, and the appropriate radon-specific regulatory benchmark is the reference level of 300 Bq/m^3^ (1, 4). All annual dose estimates are presented in mSv/y and WLM/y only.

## 6 Discussion

### 6.1 Interpretation of health risk and causality

A critical question is whether post-earthquake radon exposure plausibly translates into increased lung cancer risk in affected populations. The absence of direct epidemiological evidence does not indicate absence of risk; rather, it stems from profound methodological challenges including the multi-decade latency of lung cancer (70–72), population displacement that complicates long-term cohort studies (73, 74), and confounding from concurrent post-disaster inhalation hazards such as asbestos and silica dust (9, 72, 75).

Quantitative illustrations in this paper suggest that elite athletes training regularly in post-earthquake environments at 300–500 Bq/m^3^ may accumulate estimated annual effective doses of 3.4–8.4 mSv/y—levels that, while below occupational limits, carry meaningful incremental risk under the linear no-threshold model. Recent pooled analyses from the Pooled Uranium Miners Analysis (PUMA), focusing on miners hired in 1960 or later, confirm a clear linear exposure-response relationship even at low cumulative exposures (ERR/100 WLM = 1.33; 95% CI: 0.89–1.88) (76), underscoring that post-earthquake radon elevations in gyms should not be dismissed as inconsequential.

Our argument is not that the biological pathway from radon to lung cancer changes following an earthquake, but that the exposure conditions do. Seismic activity may lead to sustained elevations of indoor radon concentrations in specific ground-contact environments, coinciding with physiological states that markedly increase internal radiation dose. While definitive epidemiological confirmation may be infeasible within any reasonable timeframe, established principles of radon dosimetry and genotoxicity compel treatment of this scenario as a significant and actionable public health concern.

### 6.2 Lung cancer risk in physically active populations: a disproportionately exposed group

Current radon reference levels, including the WHO-recommended range of 100–300 Bq/m^3^ and the EU reference level of 300 Bq/m^3^, are derived from epidemiological studies of general residential populations and are expressed exclusively in terms of airborne concentration (1, 4). They do not account for the marked physiological differences between sedentary occupants and individuals engaged in vigorous sustained exercise. As demonstrated in Table 4, the DCF for vigorous exercise (14 nSv/(Bq·h·m^−3^)) is approximately twice that for rest (6.9 nSv/(Bq·h·m^−3^)). The direct comparison of Profiles 1 and 2 in Table 5, same hours, same concentration, different physiology, quantifies this gap concretely: at 500 Bq/m^3^, a recreational user receives approximately 60% more annual dose than a casual visitor spending identical time in the same room.

As illustrated in Tables 5, 3, an elite endurance athlete training vigorously for 800–1,200 h/y in a post-earthquake gymnasium at 500 Bq/m^3^ accumulates an estimated annual effective dose of 5.60–8.40 mSv/y. These values represent a meaningful increment above average annual natural background radiation of approximately 1.0–1.8 mSv/y from all sources (77, 78). Under the linear no-threshold model, incremental increases in cumulative radon dose are assumed to proportionally elevate lifetime lung cancer risk (19). The BEIR VI models provide the most widely accepted framework for translating cumulative radon exposure in WLM/y into excess relative risk of lung cancer (19, 73, 74, 79–82). The annual WLM/y values estimated for elite athletes in Table 5 are within the range where the PUMA linear relationship applies, and cumulative exposure over a multi-year athletic career would accumulate proportionally (76).

Several characteristics of the physically active population further amplify individual risk. Many athletes begin intensive indoor training during adolescence or early adulthood, meaning exposure begins decades earlier than typical residential exposure ages. Furthermore, athletes spend substantially more time in the affected microenvironment and do so at physiological states that maximize delivered dose per unit of time indoors. Under the UNSCEAR approach, vigorous exercise dose estimates would be approximately 35% lower, though the directional finding remains unchanged.

Table 3 reveals the most practically actionable finding of this paper: session duration and exercise intensity are the two risk factors most immediately amenable to modification during disaster recovery, requiring no structural intervention. For casual visitors, even 120-min sessions remain below 1 mSv/y at post-earthquake moderate conditions, suggesting that brief exposure restrictions focused on exercising users are more proportionate than blanket occupancy restrictions. For recreational users, reducing session duration from 90 to 30 min at 500 Bq/m^3^ reduces annual dose from approximately 1.43–2.86 mSv/y to 0.48–0.95 mSv/y, a reduction that brings most scenarios below the 1 mSv/y reference level without eliminating physical activity entirely. For elite athletes, even 30-min sessions at post-earthquake concentrations substantially exceed 1 mSv/y, indicating that temporary relocation to outdoor training is the most appropriate intervention at this level.

The evidence synthesized above supports the conclusion that frequent users of ground-floor gymnasiums in post-earthquake environments and elite athletes in particular constitute a high-risk subgroup whose lung cancer risk from radon exposure is systematically underestimated by current guidelines. This is not a consequence of uncertainty in the biological mechanisms of radon carcinogenesis, which are robustly established, but of a mismatch between the exposure assumptions embedded in existing reference levels and the physiological reality of vigorous sustained exercise.

### 6.3 Mitigation, preparedness, and regulatory policy

Given the plausible and potentially meaningful lung cancer risks identified, a proactive and integrated mitigation strategy is warranted in disaster-prone regions. Post-earthquake protocols for training facilities should include indoor radon testing using commercially available short-term measurement kits prior to resuming full-scale activities. Short-term activated charcoal detectors offer a practical and timely screening tool (83). Where elevated radon levels are confirmed, public health advisories should recommend increased ventilation and temporary restrictions on the duration or intensity of indoor strenuous activity—the two modifiable factors demonstrated in Table 3.

For new construction and major renovations in seismically active regions, radon-resistant techniques should be incorporated into building codes, including gas-permeable layers, sealed entry points, and sub-slab vent pipes creating a passive depressurization system (84, 85). Such systems continuously draw radon from beneath the foundation and vent it above the roofline, reducing indoor accumulation (86). Passive systems can be readily upgraded to active systems with the addition of a fan (1).

Sports facilities increasingly serve as temporary shelters following major earthquakes (87). When gymnasiums transition from intermittently occupied training spaces to continuously inhabited shelters, radon accumulation implications are substantially magnified, as illustrated by the extended occupancy scenarios in Table 3, Profile 1 (88). Integrating radon mitigation systems is therefore not merely an enhancement but a prerequisite for achieving dual objectives of structural safety and environmental health within a disaster preparedness framework (89).

### 6.4 Limitations

Several important limitations must be acknowledged. First, the quantitative scenarios depend on parameters, equilibrium factor, aerosol size distribution, dose conversion coefficients, that can vary significantly based on local building characteristics, ventilation, and individual physiology. Second, the post-earthquake indoor radon concentration range of 500–1,000 Bq/m^3^ involves extrapolation from soil-gas and groundwater anomaly data, as direct indoor measurements in post-earthquake gymnasiums are absent from the literature. Third, the activity-specific DCF values applied follow ICRP Publication 137 scaling (21); under the UNSCEAR approach (4), vigorous exercise dose estimates would be approximately 35% lower. Fourth, the intermediate DCF of 11 nSv/(Bq·h·m^−3^) applied to Profile 2 is an interpolated value not explicitly tabulated in ICRP 137. Fifth, the chest X-ray equivalents presented in Table 5 are provided solely for order-of-magnitude public communication and do not imply radiobiological equivalence with internal alpha irradiation.

### 6.5 Future research perspectives

Future research should address several key uncertainties identified in this study. First, longitudinal monitoring of indoor radon concentrations in ground-floor sports facilities following major seismic events is needed to determine whether the 500–1,000 Bq/m^3^ range assumed in the quantitative illustrations reflects realistic post-seismic conditions. Such measurements should combine short-term activated charcoal detectors for rapid screening with long-term alpha track detectors for sustained assessment, and should be stratified by building characteristics and proximity to active fault zones.

Second, biomarker-based studies in physically active populations exposed to elevated indoor radon levels would provide a direct empirical test of the hypothesized genotoxic burden. Validated endpoints such as micronucleus frequency, chromosomal aberrations, and DNA strand break markers could be assessed in athlete cohorts under controlled or observational conditions.

Third, epidemiological investigations linking post-disaster radon exposure to long-term lung cancer risk remain methodologically challenging but scientifically important. Integrating radon monitoring into post-disaster health surveillance systems may facilitate such studies.

Finally, the potential short-term physiological effects of radon exposure during exercise remain uncertain at environmental exposure levels. While chronic low-dose alpha irradiation under conditions of elevated ventilation may plausibly contribute to oxidative stress or genomic instability, current evidence is insufficient to support definitive conclusions regarding impacts on performance, recovery, or immune function. These questions should be considered exploratory and require controlled experimental validation using combined dosimetric and biomarker-based approaches.

## 7 Conclusion

Post-earthquake radon accumulation in ground-floor gymnasiums may represent a plausible and under-recognized environmental health concern in seismically active regions that warrants serious consideration in disaster recovery planning. By integrating geophysical evidence of earthquake-enhanced radon flux, exercise-induced amplification of respiratory dose, and established lung cancer risk models including BEIR VI, this paper identifies a plausible synergistic threat: a potentially elevated lung cancer risk for frequent gym users and athletes who train in post-seismic indoor environments with sustained radon elevations.

Quantitative illustrations grounded in established ICRP dosimetric models suggest that post-seismic radon elevations combined with vigorous exercise can amplify per-session effective dose approximately 2-fold relative to a sedentary occupant at the same concentration, and up to 40-fold relative to pre-earthquake background conditions when the combined effect of elevated concentration and exercise physiology is considered. Under these modeled conditions, elite athletes may accumulate estimated annual effective doses of 5.60–8.40 mSv/y at 500 Bq/m^3^ and 11.20–16.80 mSv/y at 1000 Bq/m^3^. These estimates are illustrative and subject to the uncertainties described in Section 2, but their direction and order of magnitude are consistent across plausible parameter ranges.

These findings suggest that standard concentration-based radon guidelines may underestimate risk for highly active populations in post-earthquake environments because they do not account for the physiological amplification of dose during vigorous exercise. Session duration and exercise intensity are immediately modifiable risk factors, as demonstrated in Table 3, and temporary activity-specific exposure guidance represents a proportionate first-line response pending structural mitigation.

Practical mitigation measures include: rapid post-earthquake radon monitoring in high-occupancy ground-level buildings using short-term detectors; targeted education for sports federations, gym operators, and public health agencies; and incorporation of radon-resistant construction techniques such as passive sub-slab depressurization systems into updated seismic building codes.

## Data Availability

This manuscript does not contain original data. No datasets were generated or analyzed in the course of this study. All quantitative illustrations are derived from published models, established dose conversion frameworks, and documented exposure parameters cited in the text.

## References

[1] World Health Organization. WHO Handbook on Indoor radon: a public Health Perspective. Geneva: WHO (2009). 23762967

[2] Baltrocchi APD Maggi L Lago BD Torretta V Szabo M Nasirov M . Mechanisms of diffusion of radon in buildings and mitigation techniques. Sustainability. (2023) 16:324. doi: 10.3390/su16010324

[3] U.S. Environmental Protection Agency. A Citizen's Guide to Radon. Washington, DC: U.S. EPA (2016).

[4] United Nations Scientific Committee on the Effects of Atomic Radiation. (2020). Lung cancer from exposure to radon (Scientific Annex B) UNSCEAR 2019 Report

[5] Appleton JD Miles JCH. A statistical evaluation of the geogenic controls on indoor radon concentrations and radon risk. J Environ Radioact. (2010) 101:799–803. doi: 10.1016/j.jenvrad.2009.06.002, 19577346

[6] Kashkinbayev Y Kazhiyakhmetova B Altaeva N Bakhtin M Tarlykov P Saifulina E . Radon exposure and cancer risk: assessing genetic and protein markers in affected populations. Biology. (2025) 14:506. doi: 10.3390/biology14050506, 40427695 PMC12109156

[7] Barberio MD Gori F Barbieri M Billi A Casalati F Franchini S . Optimization of dissolved radon monitoring in groundwater to contribute to the evaluation of the seismic activity. SN Appl Sci. (2020) 2:1392. doi: 10.1007/s42452-020-3185-2

[8] Sahoo BK Sapra BK Khan A Ratheesh MP Kumbhar DH Gaware JJ . A persistent radon anomaly signal preceding the destructive 7.7 mw earthquake in Myanmar on March 28, 2025. Sci Rep. (2025) 15:25447. doi: 10.1038/s41598-025-10632-8, 40659764 PMC12260088

[9] Balbay EG Kayalar O Balbay O Dikensoy O Arbak P Bayram H. Impact of earthquakes on lung health. Thorac Res Pract. (2024) 25:89–98. doi: 10.5152/ThoracResPract.2024.23059, 38454205 PMC11114252

[10] Hwang HS Hamm SY Cheong JY Lee SH Ha K Lee C . Effective time- and frequency-domain techniques for interpreting seismic precursors in groundwater level fluctuations on Jeju Island, Korea. Sci Rep. (2020) 10:7866. doi: 10.1038/s41598-020-64586-0, 32398830 PMC7217890

[11] Kawabata K Sato T Takahashi HA Tsunomori F Hosono T Takahashi M . Changes in groundwater radon concentrations caused by the 2016 Kumamoto earthquake. J Hydrol. (2020) 584:124712. doi: 10.1016/j.jhydrol.2020.124712

[12] Wasserman K Hansen JE Sue DY Stringer WW Sietsema KE Sun XG . Principles of Exercise Testing and Interpretation. 5th ed. Philadelphia: Lippincott Williams & Wilkins (2012).

[13] Bruce RM. The control of ventilation during exercise: a lesson in critical thinking. Adv Physiol Educ. (2017) 41:539–47. doi: 10.1152/advan.00086.2017, 29066604

[14] Niinimaa V Cole P Mintz S Shephard RJ. The switching point from nasal to oronasal breathing. Respir Physiol. (1980) 42:61–71. doi: 10.1016/0034-5687(80)90104-9, 7444224

[15] Bertoni G El Hajj TM Gandolla M. Radon risk assessment and mitigation deadlines. J Eur Radon Assoc. (2022) 3, 1–11. doi: 10.35815/radon.v3.7790

[16] Papenfuss F Maier A Sternkopf S Fournier C Kraft G Friedrich T. Radon progeny measurements in a ventilated filter system to study respiratory-supported exposure. Sci Rep. (2023) 13:10792. doi: 10.1038/s41598-023-39546-z, 37402813 PMC10319858

[17] Marsh JW Tomasek L Laurier D Harrison JD. Effective dose coefficients for radon and progeny: a review of ICRP and UNSCEAR values. Radiat Prot Dosim. (2021) 195:1–20. doi: 10.1093/rpd/ncab052 34278430

[18] Linell J Isaxon C Olsson B Londahl J Wierzbicka A Pagels J . Effects of breathing variables on modelled particle lung deposition at physical activity for children and adults. Air Qual Atmos Health. (2024) 17:843–56. doi: 10.1007/s11869-023-01484-0

[19] National Research Council. Health Effects of Exposure to Radon: BEIR VI. Washington, DC: National Academies Press (1999). 25121310

[20] International Commission on Radiological Protection. The 2007 recommendations of the international commission on radiological protection. ICRP publication 103. Ann ICRP. (2007) 37:1–332. doi: 10.1016/j.icrp.2007.10.003 18082557

[21] International Commission on Radiological Protection. Occupational intakes of radionuclides: part 3. ICRP publication 137. Ann ICRP. (2017) 46:1–486. doi: 10.1177/0146645317734963 29380630

[22] Dempsey JA Wagner PD. Exercise-induced arterial hypoxemia. J Appl Physiol. (1999) 87:1997–2006. doi: 10.1152/jappl.1999.87.6.1997 10601141

[23] Johnson BD Saupe KW Dempsey JA. Mechanical constraints on exercise hyperpnea in endurance athletes. J Appl Physiol. (1992) 73:874–86. doi: 10.1152/jappl.1992.73.3.874, 1400051

[24] Dempsey JA Romer L Rodman J Miller J Smith C. Consequences of exercise-induced respiratory muscle work. Respir Physiol Neurobiol. (2006) 151:242–50. doi: 10.1016/j.resp.2005.12.015, 16616716

[25] Rowell LB. Human Cardiovascular Control. New York: Oxford University Press (1993).

[26] Wagner PD. Determinants of maximal oxygen transport and utilization. Annu Rev Physiol. (1996) 58:21–50. doi: 10.1146/annurev.ph.58.030196.000321 8815793

[27] Swift DL Proctor DF. "Access of air to the respiratory tract". In: Brain JD Proctor DF Reid LM, editors. Respiratory Defense Mechanisms. New York: Marcel Dekker (1977). p. 63–93.

[28] Heyder J Gebhart J Rudolf G Schiller CF Stahlhofen W. Deposition of particles in the human respiratory tract in the size range 0.005-15 μm. J Aerosol Sci. (1986) 17:811–25. doi: 10.1016/0021-8502(86)90035-2

[29] Riudavets M Herreros MG de Besse B Mezquita L (2022) Radon and lung cancer: current trends and future perspectives Cancers (Basel) 14:3142 doi: 10.3390/cancers14133142 35804914 PMC9264880

[30] Shqair AA Kim E. Multi-scaled Monte Carlo calculation for radon-induced cellular damage in the bronchial airway epithelium. Sci Rep. (2021) 11:10230. doi: 10.1038/s41598-021-89689-0, 33986410 PMC8119983

[31] Danforth JM Provencher L Goodarzi AA. Chromatin and the cellular response to particle radiation-induced oxidative and clustered DNA damage. Front Cell Dev Biol. (2022) 10:910440. doi: 10.3389/fcell.2022.910440, 35912116 PMC9326100

[32] Zolghadri S Rafiepour P Yousefnia H. Quantifying DNA strand breaks from targeted alpha emitters via Geant4-DNA. EJNMMI Phys. (2025) 12:92. doi: 10.1186/s40658-025-00805-9, 41144098 PMC12559532

[33] Autsavapromporn N De Toledo SM Little JB Jay-Gerin JP Harris AL Azzam EI . The role of gap junction communication and oxidative stress in the propagation of toxic effects among high-dose alpha-particle-irradiated human cells. Radiat Res. (2011) 175:347–57. doi: 10.1667/rr2372, 21388278 PMC3139025

[34] Chen Z Li Y Liu Z Wang J Zhou X Du J. Radon emission from soil gases in the active fault zones in the capital of China and its environmental effects. Sci Rep. (2018) 8:16772. doi: 10.1038/s41598-018-35262-1, 30425320 PMC6233208

[35] Koike K Yoshinaga T Ueyama T Asaue H. Increased radon-222 in soil gas because of cumulative seismicity at active faults. Earth Planets Space. (2014) 66:57. doi: 10.1186/1880-5981-66-57

[36] Neri M Ferrera E Giammanco S Currenti G Cirrincione R Patane G . Soil radon measurements as a potential tracer of tectonic and volcanic activity. Sci Rep. (2016) 6:24581. doi: 10.1038/srep24581, 27079264 PMC4832328

[37] Omori Y Nagahama H Yasuoka Y Muto J. Radon degassing triggered by tidal loading before an earthquake. Sci Rep. (2021) 11:4092. doi: 10.1038/s41598-021-83499-0, 33603007 PMC7892827

[38] Conti L Picozza P Sotgiu A. A critical review of ground based observations of earthquake precursors. Front Earth Sci. (2021) 9:676766. doi: 10.3389/feart.2021.676766

[39] Diaz F Liza R. Radon anomalies and earthquake prediction: trends and research hotspots in the scientific literature. Geosciences. (2025) 15:283. doi: 10.3390/geosciences15080283

[40] Singh S Jaishi HP Tiwari RP Tiwari RC. A study of variation in soil gas concentration associated with earthquakes near indo-Burma subduction zone. Geoenviron Disasters. (2016) 3:22. doi: 10.1186/s40677-016-0055-8

[41] Zafar WA Ahmed J Barkat A Nabi A Mahmood R Manzoor S . Spatial mapping of radon: implication for fault delineation. Geochem J. (2018) 52:359–71. doi: 10.2343/geochemj.2.0526

[42] Woith H. Radon earthquake precursor: a short review. Eur Phys J Spec Top. (2015) 224:611–27. doi: 10.1140/epjst/e2015-02395-9

[43] D'Incecco S Petraki E Priniotakis G Papoutsidakis M Yannakopoulos P Nikolopoulos D. CO2 and radon emissions as precursors of seismic activity. Earth Syst Environ. (2021) 5:655–66. doi: 10.1007/s41748-021-00229-2

[44] Nunes LJ Curado A Lopes SI. The relationship between radon and geology: sources, transport and indoor accumulation. Appl Sci. (2023) 13:7460. doi: 10.3390/app13137460

[45] Rathebe PC Mphaga KV Masekameni DM. Climate change and environmental radioactivity: a review of studies on climate conditions in variation on indoor radon concentrations. Environ Monit Assess. (2025) 197:446–26. doi: 10.1007/s10661-025-13889-8, 40113619 PMC11926057

[46] Kim JW Joo HY Kim R Moon JH. Investigation of the relationship between earthquakes and indoor radon concentrations at a building in Gyeongju, Korea. Nucl Eng Technol. (2018) 50:512–8. doi: 10.1016/j.net.2017.12.010

[47] Goto M Yasuoka Y Nagahama H Muto J Omori Y Ihara H . Anomalous changes in atmospheric radon concentration before and after the 2011 northern Wakayama earthquake (Mj 5.5). Radiat Prot Dosim. (2017) 174:412–8. doi: 10.1093/rpd/ncw142 PMC5423104 27412515

[48] Iwata D Nagahama H Muto J Yasuoka Y. Non-parametric detection of atmospheric radon concentration anomalies related to earthquakes. Sci Rep. (2018) 8:13028. doi: 10.1038/s41598-018-31341-5, 30158564 PMC6115410

[49] Maier A Bailey T Hinrichs A Lerchl S Newman RT Fournier C . Experimental setups for in vitro studies on radon exposure in mammalian cells. Int J Environ Res Public Health. (2023) 20:5670. doi: 10.3390/ijerph20095670 37174189 PMC10178159

[50] Harrison JD Marsh JW. ICRP recommendations on radon. Ann ICRP. (2020) 49:68–76. doi: 10.1177/0146645320931974, 32746607

[51] Mahaney BL Meek K Lees-Miller SP. Repair of ionizing radiation-induced DNA double-strand breaks by non-homologous end-joining. Biochem J. (2009) 417:639–50. doi: 10.1042/bj20080413, 19133841 PMC2975036

[52] Radhakrishnan SK Jette N Lees-Miller SP. Non-homologous end joining: emerging themes and unanswered questions. DNA Repair (Amst). (2014) 17:2–8. doi: 10.1016/j.dnarep.2014.01.009, 24582502 PMC4084493

[53] Rodgers K McVey M. Error-prone repair of DNA double-strand breaks. J Cell Physiol. (2016) 231:15–24. doi: 10.1002/jcp.25053, 26033759 PMC4586358

[54] Wilson TE Sunder S. Double-strand breaks in motion: implications for chromosomal rearrangement. Curr Genet. (2020) 66:1–6. doi: 10.1007/s00294-019-01015-4, 31321486 PMC6980467

[55] Mladenov E Kallies M Stuschke M Gkika E Iliakis G. CRISPR/Cas9 generated DSB clusters mimic complex lesions induced by high-LET radiation. Sci Rep. (2025) 15:36480. doi: 10.1038/s41598-025-22945-9, 41115973 PMC12537819

[56] Takahashi A Kubo M Ma H Nakagawa A Yoshida Y Isono M . Nonhomologous end-joining repair plays a more important role than homologous recombination repair in defining radiosensitivity after exposure to high-LET radiation. Radiat Res. (2014) 182:338–44. doi: 10.1667/rr13782.1, 25117625

[57] Michaeli O Luz I Vatarescu M Manko T Weizman N Korotinsky Y . APR-246 as a radiosensitization strategy for mutant p53 cancers treated with alpha-particles-based radiotherapy. Cell Death Dis. (2024) 15:426. doi: 10.1038/s41419-024-06830-3, 38890278 PMC11189442

[58] Nepomuceno LL Ferreira JL de Sousa Cruz V Gabriel GH Araujo EG. P53 protein and its fundamental role in the cell cycle, apoptosis and cancer. Encicl Biosfera. (2018) 15:760–78. doi: 10.18677/EnciBio_2018B64

[59] Vaddavalli PL Schumacher B. The p53 network: cellular and systemic DNA damage responses in cancer and aging. Trends Genet. (2022) 38:598–612. doi: 10.1016/j.tig.2022.02.010, 35346511

[60] Smerhovsky Z Landa K Rossner P Brabec M Zudova Z Hola N . Increased risk of cancer in radon-exposed miners with elevated frequency of chromosomal aberrations. Radiat Res. (2002) 157:585–93. doi: 10.1667/0033-7587(2002)157[0585,IROCIR]2.0.CO;2 11815255

[61] Bersimbaev R Bulgakova O. "Residential radon exposure and lung cancer risk in Kazakhstan". In: Neznal M, editor. Radon. London: IntechOpen (2017)

[62] Vahäkangas KH Bennett WP Castren K Welsh JA Khan MA Blomeke B . P53 and K-Ras mutations in lung cancers from former and never-smoking women. Cancer Res. (2001) 61:4350–6. 11389059

[63] Popp W Vahrenholz C Schuster H Wiesner B Bauer P Tauscher F . P53 mutations and codon 213 polymorphism of P53 in lung cancers of former uranium miners. J Cancer Res Clin Oncol. (1999) 125:309–12. doi: 10.1007/s004320050279, 10359137 PMC12199856

[64] Bridges BA Cole J Arlett CF Green MHL Waugh APW Beare D . Possible association between mutant frequency in peripheral lymphocytes and domestic radon concentrations. Lancet. (1991) 337:1187–9. doi: 10.1016/0140-6736(91)92859-Z 1673739

[65] Chen Z Wang D Gu C Liu X Pei W Li J . Down-regulation of let-7 microRNA increased K-Ras expression in lung damage induced by radon. Environ Toxicol Pharmacol. (2015) 40:541–8. doi: 10.1016/j.etap.2015.08.009, 26318567

[66] Shanahan EM Peterson D Roxby D Quintana J Morely AA Woodward A. Mutation rates at the glycophorin a and HPRT loci in uranium miners exposed to radon progeny. Occup Environ Med. (1996) 53:439–44. doi: 10.1136/oem.53.7.439 8704866 PMC1128510

[67] Dicu T Virag P Brie I Perde-Schrepler M Fischer-Fodor E Victor B . A comparative study of genotoxicity endpoints for women exposed to different levels of indoor radon concentrations. Int J Radiat Biol. (2021) 98:18. doi: 10.1080/09553002.2021.1987559, 34586971

[68] Hellman B Friis L Vaghef H Edling C. Alkaline single cell gel electrophoresis and human biomonitoring for genotoxicity: a study on subjects with residential exposure to radon. Mutat Res. (1999) 442:121–32. doi: 10.1016/s1383-5718(99)00083-2, 10393281

[69] Azzam EI Jay-Gerin JP Pain D. Ionizing radiation-induced metabolic oxidative stress and prolonged cell injury. Cancer Lett. (2012) 327:48–60. doi: 10.1016/j.canlet.2011.12.012, 22182453 PMC3980444

[70] Zablotska LB Richardson DB Golden A Pasqual E Smith B Rage E . The epidemiology of lung cancer following radiation exposure. Int J Radiat Biol. (2023) 99:569–80. doi: 10.1080/09553002.2022.2110321, 35947399 PMC9943789

[71] Antignani S Venoso G Ampollini M Caprio M Carpentieri C Di Carlo C . A 10-year follow-up study of yearly indoor radon measurements in homes and implications on lung cancer risk estimates. Sci Total Environ. (2021) 762:144150. doi: 10.1016/j.scitotenv.2020.144150 33418274

[72] Oral T Ozay ME Ucan R Aker D Can E Altinten B. Measurement and evaluation of dust concentrations in the air after the Kahramanmaras earthquake in Turkey. Int J Environ Res Public Health. (2025) 22:649. doi: 10.3390/ijerph22040649, 40283870 PMC12027378

[73] Abramson D Stehling-Ariza T Garfield R Redlener I. Prevalence and predictors of mental health distress post-Katrina. Disaster Med Public Health Prep. (2008) 2:77–86. doi: 10.1097/dmp.0b013e318173a8e7 18520693

[74] Yamamoto K Takita M Kami M Tani Y Yamamoto C Zhao T . Loss of participation among evacuees aged 20-37 years in the disaster cohort study after the great East Japan earthquake. Sci Rep. (2022) 12:19600. doi: 10.1038/s41598-022-23896-1, 36380078 PMC9665037

[75] Li J Cone JE Kahn AR Brackbill RM Farfel MR Greene CM . Association between world trade center exposure and excess cancer risk. JAMA. (2012) 308:2479–88. doi: 10.1001/jama.2012.110980, 23288447

[76] Richardson DB Rage E Demers PA Do MT Fenske N Deffner V Lung cancer and radon: pooled analysis of uranium miners hired in 1960 or later. Environ Health Perspect (2022);130:57010. doi:doi: 10.1289/EHP10669 35604341 PMC9126132

[77] United Nations Scientific Committee on the Effects of Atomic Radiation (2008) Sources and Effects of Ionizing Radiation: UNSCEAR 2008 Report, Vol I, United Nations

[78] Porstendorfer J. Influence of physical parameters on doses from radon exposures. In: International Congress Series. Vol 1225. Elsevier; (2002). 149–160.

[79] Ruano-Ravina A Martin-Gisbert L Kelsey K Pérez-Ríos M Candal-Pedreira C Rey-Brandariz J . An overview on the relationship between residential radon and lung cancer: what we know and future research. Clin Transl Oncol. (2023) 25:3357–68. doi: 10.1007/s12094-023-03308-0, 37610496 PMC10603006

[80] Fang L Wu F Sun H Li W Hou D Ma Y. Analysis of genetic instability induced by radon exposure in iron mine processing workers in Shandong Province, northern China. Front Public Health. (2024) 12:1452730. doi: 10.3389/fpubh.2024.1452730, 39735759 PMC11681618

[81] Robertson A Allen J Laney R Curnow A. The cellular and molecular carcinogenic effects of radon exposure: a review. Int J Mol Sci. (2013) 14:14024–63. doi: 10.3390/ijms140714024, 23880854 PMC3742230

[82] Muller WU Giussani A Ruhm W Lecomte JF Harrison J Kreuzer M . Current knowledge on radon risk: implications for practical radiation protection? radon workshop, 1/2 December 2015, Bonn, BMUB (Bundesministerium für Umwelt, Naturschutz, Bau und Reaktorsicherheit; Federal Ministry for the Environment, Nature Conservation, Building and Nuclear Safety). Radiat Environ Biophys. (2016) 55:267–80. doi: 10.1007/s00411-016-0657-2 27334644 PMC4951500

[83] Mphaga KV Mbonane TP Utembe W Rathebe PC. Short-term vs. long-term: a critical review of indoor radon measurement techniques. Sensors (Basel). (2024) 24:4575. doi: 10.3390/s24144575, 39065973 PMC11280955

[84] Monahan E Murphy P Long S Dowdall A. The effectiveness of passive sumps and static cowls in reducing radon levels in new build Irish dwellings. J Environ Radioact. (2022) 248:106866. doi: 10.1016/j.jenvrad.2022.106866, 35358917

[85] Jelle BP. Development of a model for radon concentration in indoor air. Sci Total Environ. (2012) 416:343–50. doi: 10.1016/j.scitotenv.2011.11.052, 22178027

[86] Fuente M Rabago D Goggins J Fuente I Sainz C Foley M. Radon mitigation by soil depressurisation case study. Sci Total Environ. (2019) 695:133746. doi: 10.1016/j.scitotenv.2019.133746 31416037

[87] Wu Y Zhao L Sun J. Analysis of the emergency shelter function of large-scale international sports event venues. Transp Res Interdiscip Perspect. (2025) 34:101680. doi: 10.1016/j.trip.2025.101680

[88] Cao X Fang H Yuan X. Toward health-oriented indoor air quality in sports facilities: a narrative review of pollutant dynamics, smart control strategies, and energy-efficient solutions. Buildings. (2025) 15:3168. doi: 10.3390/buildings15173168

[89] Gebel S Koc D Ayci H. Spor komplekslerinin deprem sonrasi kullanimi ve potansiyelleri: Kahramanmaras ve Gaziantep ornekleri. J Archit Sci Appl. (2023) 8:198–221. doi: 10.30785/mbud.1334040

[90] Wang J He L Fan D Ding D Wang X Gao Y . Establishment of a γ-H2AX foci-based assay to determine biological dose of radon to red bone marrow in rats. Sci Rep. (2016) 6:30018. doi: 10.1038/srep30018, 27445126 PMC4957115

[91] Lehnert BE Goodwin EH Deshpande A. Extracellular factor(s) following exposure to alpha particles can cause sister chromatid exchanges in normal human cells. Cancer Res. (1997) 57:2164–71. 9187116

[92] Weaver D. Cytogenetic and molecular genetic analysis of tumorigenic human bronchial epithelial cells induced by radon alpha particles. Carcinogenesis. (1997) 18:1251–7. doi: 10.1093/carcin/18.6.1251, 9214610

[93] Paolicchi F Miniati F Bastiani L Faggioni L Ciaramella A Creonti I . Assessment of radiation protection awareness and knowledge about radiological examination doses among Italian radiographers. Insights Imaging. (2016) 7:233–42. doi: 10.1007/s13244-015-0445-6, 26596570 PMC4805619

